# Prevalence of participation of Brazilian aged people in Advanced Activities of Daily Living and associated factors

**DOI:** 10.1590/1980-549720240070

**Published:** 2024-12-16

**Authors:** Vanessa de Barros e Silva Mazer, Rafael da Silveira Moreira, Kenio Costa de Lima, Maria das Graças Wanderley de Sales Coriolano, Vanessa de Lima Silva

**Affiliations:** I Universidade Federal de Pernambuco – Recife (PE), Brazil.; II Universidade Federal do Rio Grande do Norte – Natal (RN), Brazil.

**Keywords:** Activities of daily living, Aged, Leisure activities, Social participation, Work, Functional status

## Abstract

**Objective:**

To estimate the prevalence of participation of aged Brazilians in Advanced Activities of Daily Living and associated factors.

**Methods:**

Cross-sectional study developed with secondary data from the National Health Survey. Aged people (60 years old or older) were included. The dependent variable consisted of questions from the National Health Survey regarding the performance of Advanced Activities of Daily Living, related to work, leisure, and social participation. Descriptive analysis, Rao-Scott test, and standardized residual analysis were performed. Effect measures were calculated using simple and multiple multinominal logistic regression models.

**Results:**

The majority of Brazilian aged people have low participation in Advanced Activities of Daily Living. Aged males, between 60 and 69 years old, white, from the Southeast, South and Central-West regions, with higher educational levels, absence of chronic diseases, monthly alcohol consumption, regular physical activity, frequent intake of vegetables, use of glasses and independence when moving, were more likely to perform Advanced Activities of Daily Living.

**Conclusion:**

These results reinforce the significant influence of demographic, socioeconomic, clinical factors, linked to lifestyle and intrinsic capacity in the execution of Advanced Activities of Daily Living. In a broader sense, such discoveries can strengthen public policies for active and healthy aging.

## INTRODUCTION

Performing Activities of Daily Living (ADLs) is essential for maintaining the physical, mental, and social capacities of the aged and serves as a valuable measure for assessing functionality^1,2^. ADLs are categorized into Basic Activities of Daily Living (BADLs), Instrumental Activities of Daily Living (IADLs), and Advanced Activities of Daily Living (AADLs^3^.

AADLs refer to physical and social functions performed voluntarily in everyday life. While these activities are not exclusive to older adults, this age group tends to experience a greater decline in AADLs due to changes in overall health, as well as in cognitive and social capacities^4^.

In 1989, Reuben and Solomon categorized AADLs into activities related to social integration, productivity, and leisure^5^. Impairments in these areas may precede functional limitations in IADLs and BADLs^6^.

The performance of AADLs is directly linked to healthy aging, which encompasses the development and maintenance of functional capacity and contributes to well-being in older age^7,8^. Consequently, preserving functional independence throughout life is a primary objective, positioning functional status as a key paradigm in understanding the health of aged individuals^9^.

Although AADLs may seem familiar, they are addressed differently in the literature. Many authors do not distinguish between AADLs and IADLs, instead grouping them together as ADLs. Others treat AADLs as a separate category, subdividing them into leisure activities, social participation activities, and work-related activities.

In the Brazilian context, alongside these gaps, there is a lack of analysis regarding factors influencing the performance of AADLs. A 2020 study in Brazil evaluated the number of AADLs performed by older adults from a list of 13 items^10^. Another study, conducted by Castro et al.^11^, examined the participation of older Brazilians in paid work activities.

A 2021 study conducted in Brazil focused on the participation of older adults in social activities^12^. Additionally, Usnayo et al.^13^ in Brazil identified factors that negatively affect the participation of older adults in social and leisure activities.

Understanding the factors that may influence AADLs among aged Brazilians remains limited in the literature, as most studies have focused on localized samples or on isolated aspects of AADLs (leisure, work, or social participation). Therefore, investigating the factors that impact the performance of these activities is essential.

In this context, this research is particularly relevant as it helps address the gap in Brazilian literature on AADLs. Additionally, it holds significant scientific value by providing an overview of the functional capacity of older adults in Brazil prior to the COVID-19 pandemic, establishing a basis for comparison in future studies.

The objective of this study was to identify the prevalence of participation of aged Brazilians in AADLs and the factors associated with this participation, using data from the 2019 National Health Survey (*Pesquisa Nacional de Saúde* – PNS).

## METHODS

A cross-sectional, population-based study was conducted using an analytical approach to secondary data from the 2019 PNS database. The target population of PNS comprises residents of permanent private households across the entire national territory. For this study, data from the PNS related to individuals aged 60 years old and older were utilized.

The sampling plan employed by PNS utilizes a three-stage cluster sampling approach, with stratification of Primary Sampling Units (PSUs). The first-stage PSUs correspond to census sectors or groups of sectors, the second-stage PSUs are households, and the third-stage PSUs are residents aged 15 years old or older. The selection of units at each stage was performed using Simple Random Sampling (SRS).

The PNS-IBGE database is publicly available on the IBGE website (http://www.ibge.gov.br) and was downloaded and organized in a statistical program for data analysis. The PNS 2019 project received approval from the National Research Ethics Committee (*Comissão Nacional de Ética em Pesquisa* – CONEP) of the National Health Council (*Conselho Nacional de Saúde* – CNS) in August 2019 under approval number 3.529.376 and adhered to Resolution 196/96 of the National Health Council.

The dependent variable of this study is AADLs. The list of AADLs, which includes social, productive, and leisure activities, was adapted from the Frailty in Aged Brazilians (*Fragilidade em Idosos Brasileiros* – FIBRA) study as the basis for selecting the variables^14^.

The variables related to AADLs were selected from the PNS database based on the items from the AADL list in the FIBRA protocol. Six variables that align with AADLs were identified: participation in collective religious activities; participation in social gatherings for physical, recreational, or artistic activities; driving a car; volunteer work; paid work; and participation in associations or social movements (Supplementary Chart 1).

Given that there is no validated scale to measure the phenomenon of AADLs and that the PNS questionnaire includes six questions related to AADLs, the analysis was conducted using the statistical method of Latent Class Analysis (LCA).

The independent variables were classified into the following categories: demographic variables (gender, age, race/color, marital status, and regions); socioeconomic variables (education and place of residence); clinical variables (self-assessment of health according to the World Health Organization – WHO, falls, depression, cancer, chronic kidney disease, circulatory system diseases, endocrine system diseases, respiratory system diseases, and musculoskeletal system diseases); lifestyle-related variables (alcohol consumption, physical activity, tobacco use, consumption of vegetables or legumes, consumption of fruits, consumption of soft drinks, and consumption of industrialized sweet foods); and variables related to intrinsic capacity (vision, hearing, and locomotion).

In LCA, the optimal number of classes to best define the study's object was determined using several statistical criteria: entropy, the Akaike Information Criterion (AIC), the Bayesian Information Criterion (BIC), and the adjusted BIC. These criteria were used to evaluate model fit through the statistical program Mplus 8.

To evaluate the progression of the testing model, the Vuong-Lo-Mendell-Rubin likelihood ratio test (VLMR) and the Lo-Mendell-Rubin likelihood ratio test (LMR) were used, with p-values <0.05 considered statistically significant. Five models were tested for this study, corresponding to two, three, four, five, and six latent classes.

The data were analyzed using a statistical program that accounted for the PNS sample weights and strata. Variables with more than five categories were restructured. Descriptive analysis included absolute and relative frequencies, and the 95% confidence interval (CI) was calculated. The association between the independent variables and the dependent variable was then examined using the Rao-Scott test for complex samples and standardized residual analysis. A significance level of 5% was adopted.

The effects of the factors on the dependent variable were measured using simple and multiple models of multinomial logistic regression, following the hierarchical approach proposed by Victora. To stratify the hierarchical levels, the Social Determinants of Health (SDH) model by Dahlgren and Whitehead was used. Variables with a p-value <0.25 in the simple analysis were selected for inclusion in the multiple analysis.

## RESULTS

Out of 43,554 aged individuals in the PNS database, only 22,728 responded to the resident questionnaire and were included in this study. The frequencies of the independent variables are presented in Table 1. Most aged individuals were from the Northeast (34%). The predominant age group was 60 to 69 years (55.2%), with 55.2% being female and 43.8% married. The majority resided in private homes (83.3%), had completed elementary school (48.9%), and self-identified as either brown (44%) or white (43.6%).

**Table 1:** Descriptive and analytical analysis of Advanced Activities of Daily Living according to demographic, socioeconomic, clinical, lifestyle, and intrinsic capacity variables. National Health Survey (*Pesquisa Nacional de Saúde*), 2023.

Characteristic		High participation level in AADL% (95%CI)	Intermediate participation level in AADL% (95%CI)	Low participation level in AADL% (95%CI)	p-value	Total %
**Demographic variables**						
Gender						
	Male	82 (79–84.6)^a^	49.9 (47.6–52.1)^a^	37.6 (36.4–38.8)	<0.001*	44.8
	Female	18 (15.4–21)	50.1 (47.9–52.4)	62.4 (61.2–63.6)^a^		55.2
Age (years)						
	60 to 69	75 (72–77.8)^a^	66.7 (64.4–68.9)^a^	48.7 (87.5–49.9)	<0.001*	55.2
	70 to 79	21.4 (18.7–24.3)	26.9 (24.9–29.1)	33.6 (32.5–34.7)^a^		31.5
	80 or +	3.6 (2.1–5.1)	6.4 (5.4–7.5)	17.7 (16.7–18.7)^a^		13.3
Race/color						
	White	68 (64.7–71.3)^a^	60.4 (58–62.6)^a^	47.5 (46.2–48.8)	<0.001*	43.6
	Black	29.2 (26.3–32.4)	38 (35.8–40.3)	50.7 (49.4–52)^a^		54.8
	Yellow/indigenous	2.7 (1.7–4.1)	1.7 (1.1–2.5)	1.8 (1.5–2.2)		1.6
Marital status						
	Married	59.7 (55.6–63.6)^a^	52.4 (50.1–54.7)^a^	38.6 (37.5–39.8)	<0.001*	43.8
	Divorced	17.8 (14.6–21.5)^a^	12.6 (11.2–14.2)	10.3 (9.6–11.1)		10.9
	Widowed	9.8 (8–12.1)	21.6 (19.7–23.6)	33 (31.8–34.1)^a^		26.8
	Single	12.7 (10.7–15)	13.4 (11.9–14.9)	18.1 (17.1–19)^a^		18.5
Regions						
	North	3.1 (2.6–3.8)	4.1 (3.7–4.7)	5.7 (5.4–6)^a^	<0.001*	15.3
	Northeast	13.6 (11.7–15.7)	16.6 (15.2–18.2)	27.6 (26.7–28.6)^a^		34
	Southeast	56.6 (53.1–60.1)	47.2 (44.7–49.7)^a^	46.6 (45.4–47.9)		25.6
	South	19.7 (17.4–22.2)^a^	25.1 (23.2–27.1)^a^	14.4 (13.7–15.2)		14.6
	Central-West	7 (6–8.2)	7 (6.1–7.9)	5.6 (5.3–6)		10.4
**Socioeconomic variables**						
Education						
	No schooling	0.2 (0.0–0.7)	0.1 (0.0–0.2)	0.6 (0.5–0.8)^a^	<0.001*	0.6
	Literate	0.9 (0.5–1.6)	2.8 (2.2–3.7)	6.6 (6–7.2)^a^		6.1
	Elementary school	42.8 (39.4–46.2)	43.8 (41.1–46.4)	68.1 (66.8–69.4)^a^		48.9
	High school	24.7 (21.8–28)^a^	23.6 (21.5–25.7)^a^	16.5 (15.5–17.5)		15.7
	Higher education	31.4 (27.9–35.1)^a^	29.8 (27.3–32.3)^a^	8.2 (7.4–9)		11.8
Living situation						
	House	77.4 (73.2–81.1)	78.7 (76.3–80.9)	90.5 (89.6–91.4)^a^	<0.001*	88.3
	Apartment	22.5 (18.8–26.7)^a^	22.2 (19–23.6)^a^	9.4 (8.5–10.3)		11.5
	Living in a rooming house, shack, or slum	0.1 (0.0–0.2)	0.1 (0.0–0.3)	0.1 (0.0–0.1)		0.2
**Clinical variables**						
Self-assessed health according to the WHO						
	Very good	18.4 (15.8–21.2)^a^	19.6 (17.7–21.6)^a^	8.4 (7.7–9.2)	<0.001*	10.3
	Good	60.3 (56.6–64)^a^	58 (55.7–60.2)^a^	48.4 (47.2–49.6)		51.4
	Fair	19.1 (16.6–21.9)	19.8 (18.1–21.7)	34.3 (33.1–35.6)^a^		30.9
	Poor	2.1 (1.2–3.5)	2.4 (1.8–3.2)	6.9 (6.3–7.5)^a^		6.1
	Very poor	0.1 (0.0–0.7)	0.3 (0.1–0.6)	2 (1.7–2.4)^a^		1.3
Falls						
	Yes	7.3 (5.8–9.1)	12.1 (10.8–13.6)	18.4 (17.5–19.4)^a^	<0.001*	16.4
	No	92.7 (90.9–94.2)^a^	87.9 (86.4–89.2)^a^	81.6 (80.6–82.5)		83.6
Depression						
	Yes	8.5 (6.4–11.1)	12.1 (10.7–13.6)	12.5 (11.7–13.3)^a^	0.011	10.4
	No	91.5 (88.9–93.6)^a^	87.9 (86.4–89.3)	87.5 (86.7–88.3)		89.6
Cancer						
	Yes	6.6 (4.8–9.1)	8.8 (7.5–10.2)^a^	6.6 (6–7.2)	0.21	6.2
	No	93.4 (90.9–95.2)	91.2 (89.8–92.5)	93.4 (92.8–94)^a^		93.8
Chronic kidney disease						
	Yes	1.2 (0.8–2)	2.9 (2.2–3.9)^a^	2.4 (2.1–2.8)	0.23	2.3
	No	98.8 (98–99.2)^a^	97.1 (96.1–97.8)	97.6 (97.2–97.9)		97.7
Circulatory System Diseases						
	None	51.1 (47.9–54.3)^a^	45.7 (43.2–48.2)^a^	36.1 (35–37.3)	<0.001*	40.5
	1	40.9 (37.7–44.1)	44.6 (42.2–47)	49.3 (48.1–50.5)^a^		46.8
	2 or 3	8.1 (6.3–10.2)	9.7 (8.4–11.2)	14.5 (13.7–15.4)^a^		12
Endocrine System Diseases						
	None	67.5 (64.2–70.7)^a^	60.4 (57.9–62.9)	58.9 (57.7–60.2)	<0.001*	59.4
	1	26.7 (23.8–29.8)	33.7 (31.3–36.1)^a^	31.5 (30.4–32.7)		28.4
	2	5.8 (4.4–7.6)	5.9 (5–7.1)	9.5 (8.9–10.3)^a^		8.1
Respiratory System Diseases						
	None	94.4 (92.7–95.8)	93.8 (92.7–94.8)	92.8 (92.1–93.5)	0.135	93.9
	1	5.1 (3.8–6.8)	5.3 (4.4–6.3)	6.1 (5.5–6.9)^a^		5.4
	2	0.4 (0.2–1)	0.9 (0.6–1.4)	1 (0.8–1.3)^a^		0.8
Musculoskeletal System Diseases						
	None	71.4 (67.6–74.9)^a^	61.2 (58.9–63.5)	59.5 (58.2–60.7)	<0.001*	62.3
	1	24.6 (21.5–28)	30.8 (28.7–33)	29.4 (28.2–30.5)		28.5
	2	4 (2.8–5.6)	8 (6.9–9.3)	11.2 (10.4–12)^a^		9.2
**Lifestyle-related variables**						
Alcohol Consumption						
	Never	42.2 (38.5–46)	58.2 (55.9–60.6)	80.4 (79.3–81.4)^a^	<0.001*	75.2
	Less than once a month	10.1 (8.4–12.2)^a^	11.2 (9.9–12.7)^a^	7 (6.4–7.7)		8.3
	Once or more a month	47.7 (43.7–51.7)^a^	30.6 (28.4–32.8)^a^	12.6 (11.8–13.5)		16.5
Physical Activity						
	Yes	42.9 (39.5–46.4)^a^	59 (56.7–61.4)^a^	22.1 (21.2–23.1)	<0.001*	29.1
	No	57.1 (53.6–60.5)	41 (38.6–43.3)	77.9 (76.9–78.8)^a^		70.9
Tobacco Use						
	Yes, daily	14.6 (11.9–17.9)^a^	8.3 (7.1–9.6)	11.6 (10.9–12.4)	<0.001*	11.1
	Yes, less than daily	0.3 (0.1–0.7)	0.4 (0.2–0.8)	0.7 (0.5–1)^a^		0.7
	Currently not	85.1 (81.9–87.8)	91.3 (90–92.5)^a^	87.7 (86.8–88.4)		88.2
Vegetable or legume consumption (times per week)						
	Never	3 (2.1–4.2)	2.4 (1.7–3.2)	6.7 (6.2–7.2)^a^	<0.001*	7.6
	1 to 3	20.2 (17.7–22.9)	19.2 (17.3–21.1)	26.7 (25.6–27.8)^a^		28.4
	4 to 7	76.8 (74–79.5)^a^	78.5 (76.4–80.4)^a^	66.6 (65.5–67.7)		64
Fruit Consumption (times per week)						
	Never	7.8 (5.9–10.2)	4.7 (3.9–5.8)	7.9 (7.3–8.6)^a^	<0.001*	7.9
	1 to 3	23.6 (20.8–26.7)	20.2 (18.2–22.3)	28.7 (27.6–29.8)^a^		28.8
	4 to 7	68.6 (65.2–71.8)^a^	75.1 (72.8–77.2)	63.4 (62.2–64.5)		63.4
Soda Consumption (times per week)						
	Never	62.1 (58.8–65.4)	69.5 (67.2–71.7)	70.3 (69.1–71.5)^a^	<0.001*	72.4
	1 to 3	28.5 (25.5–31.6)^a^	24.4 (22.4–26.5)	23.6 (22.5–24.7)		22.4
	4 to 7	9.4 (7.6–11.5)^a^	6.1 (5–7.3)	6.1 (5.5–6.9)		5.2
Consumption of industrialized sweet foods (times per week)						
	Never	47.6 (44.2–50.9)	48.4 (45.9–50.8)	56.5 (55.2–57.7)^a^	<0.001*	58.2
	1 to 3	34.5 (31.2–38)^a^	34.4 (32.1–36.8)^a^	28.7 (27.6–29.8)		28.1
	4 to 7	17.9 (15.2–21)^a^	17.3 (15.5–19.1)	14.9 (14–15.8)		13.6
**Variables related to intrinsic capacity**						
Do you use glasses or other assistive devices to aid with vision problems?						
	Yes	82.2 (79.6–84.6)^a^	82.5 (80.7–84.2)^a^	70 (68.9–71.1)	<0.001*	69.9
	No	17.8 (15.4–20.4)	17.5 (15.8–19.3)	30 (28.9–31.1)^a^		30.1
Do you use hearing aids or other assistive devices to hear better?						
	Yes	3.5 (2.4–5.1)	2.9 (2.2–3.8)	3.2 (2.8–3.7)	0.68	2.8
	No	96.5 (94.9–97.6)	97.1 (96.2–97.8)	96.8 (96.3–97.2)		97.2
Do you use any assistive devices to aid mobility?						
	Yes	1.6 (0.9–2.6)	1.9 (1.4–2.7)	8.6 (7.9–9.3)^a^	<0.001*	6.2
	No	98.4 (97.4–99.1)^a^	98.1 (97.3–98.6)^a^	91.4 (90.7–92.1)		93.8

AADL: Advanced Activities of Daily Living; 95%CI: 95% confidence interval; WHO: World Health Organization.

* p<0.05 (Rao and Scott Test);

a Standardized residuals >1.96.

Source: Prepared by the author.

In terms of health, 46.8% had circulatory diseases, and 37.7% had musculoskeletal diseases, while 83.6% reported no falls in the past 12 months. Regarding lifestyle, most individuals did not consume alcohol (75.2%), did not engage in physical activity (70.9%), did not smoke (88.2%), and consumed vegetables or legumes daily (64%) as well as fruits (63.4%). Additionally, the majority did not consume soft drinks (72.4%) or industrialized sweets (58.2%).

The three-class latent model was selected due to its superior performance (Supplementary Table 1), revealing distinct patterns of engagement in AADL among the identified classes.

Based on the definition of AADL adopted in this research, along with the frequency and complexity of participation in these activities (Supplementary Graphic 1), the following categories were designated: high level of participation for Class 1, intermediate level of participation for Class 2, and low level of participation for Class 3.

The majority showed a low level of participation in AADL, accounting for 73.1%, followed by an intermediate level of participation (18.1%) and a high level of participation (8.9%).

The bivariate analysis (Table 1) revealed significant associations between AADL performance and various variables, including gender, age, race/color, marital status, region, education, self-rated health, history of falls, depression, and several chronic diseases. Additional associated factors included alcohol consumption, physical activity, smoking, dietary habits, and the use of visual correction and mobility aids.

**Figure 1:**
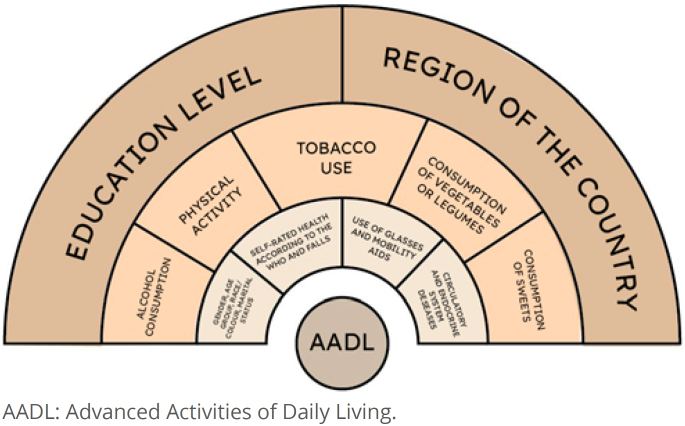
Final model of the multinomial logistic regression variables of factors associated with the performance of Advanced Activities of Daily Living in Brazilian aged individuals, 2023.

Source: Prepared by the author.

Multinomial logistic regression analysis (Table 2) was performed, including variables with p-values <0.25 in the Rao-Scott test. The associated factors that remained in the final model, organized by the hierarchical levels of SDH, are presented in Figure 1.

**Table 2:** Adjusted odds ratio values and confidence intervals obtained through multinomial logistic regression analysis for the association between demographic, socioeconomic, clinical, lifestyle, and intrinsic capacity variables and Advanced Activities of Daily Living, 2023.

Characteristic		High participation level in AADL	Intermediate participation level in AADL % (95%CI)
		OR (95%CI)	OR (95%CI)
**Demographic variables**			
Gender			
	Male	7.68 (6.12–9.64)*	1.84 (1.59–2.12)*
	Female	1.00	1.00
Age range (years)			
	60 to 69	4.46 (2.89–6.88)*	2.25 (1.77–2.84)*
	70 to 79	2.14 (1.37–3.35)*	1.54 (1.21–1.96)*
	80 or +	1.00	1.00
Race/color			
	White	1.64 (1.34–2.01)*	1.07 (0.93–1.23)
	Yellow/indigenous	1.77 (0.97–3.22)	0.78 (0.36–1.42)
	Black	1.00	1.00
Marital status			
	Married	1.25 (0.94–1.68)	1.14 (0.96–1.35)
	Divorced	1.57 (1.06–2.31)*	0.92 (0.73–1.16)
	Single	0.95 (0.66–1.36)	0.74 (0.59–0.92)*
	Widowed	1.00	1.00
Regions			
	North	1.04 (0.81–1.33)	1.20 (1.00–1.45)
	Southeast	1.57 (1.28–1.94)*	1.18 (1.01–1.37)*
	South	2.15 (1.74–2.64)*	2.55 (2.18–2.99)*
	Central-West	1.96 (1.53–2.50)*	1.13 (1.44–2.08)*
	Northeast	1.00	1.00
**Socioeconomic variables**			
Education			
	Literate	0.53 (0.12–2.26)*	5.07 (1.48–17.31)*
	Elementary school	1.71 (0.44–6.59)*	5.84 (1.76–19.34)*
	High school	4.1 (1.07–16.09)*	13.63 (4.1–45.27)*
	Higher education	10.62 (2.74–41.17)*	36.02 (10.82–119.82)*
	No schooling	1.00	1.00
**Clinical variables**			
Self-assessed health according to the WHO			
	Very good	6.37 (1.23–32.89)*	7.99 (3.02–21.24)*
	Good	5.03 (0.99–25.61)	5.82 (2.20–15.40)*
	Fair	3.53 (0.69–18.07)	4.11 (1.55–10.86)*
	Poor	2.96 (0.52–16.74)	4.08 (1.45–11.44)*
	Very poor	1.00	1.00
Falls			
	Yes	1.00	1.00
	No	1.49 (1.09–2.03)*	1.05 (0.88–1.25)
Circulatory System Diseases			
	None	1.53 (1.12–2.11)*	1.21 (0.96–1.52)
	1	1.29 (0.94–1.76)	1.05 (0.85–1.31)
	2 or 3	1.00	1.00
Endocrine System Diseases			
	None	1.17 (0.80–1.71)	1.38 (1.07–1.78)*
	1	1.25 (0.86–1.83)	1.18 (0.93–1.50)
	2	1.00	1.00
**Lifestyle-related variables**			
Alcohol Consumption			
	Never	0.21 (0.17–0.25)*	0.45 (0.39–0.53)*
	Less than once a month	0.43 (0.33–0.57)*	0.75 (0.60–0.94)*
	Once or more a month	1.00	1.00
Physical activity			
	Yes	1.69 (1.43–2.00)*	3.65 (3.24–4.11)*
	No	1.00	1.00
Tobacco Use			
	Currently not	0.87 (0.67–1.13)	1.31 (1.05–1.64)*
	Yes, less than daily	0.38 (0.14–0.99)*	0.82 (0.30–2.22)
	Yes, daily	1.00	1.00
Vegetable or legume consumption (times per week)			
	4 to 7	1.12 (0.76–1.67)	1.56 (1.07–2.27)*
	1 to 3	1.11 (0.73–1.68)	1.39 (0.93–2.08)
	Never	1.00	1.00
Consumption of industrialized sweet foods (times per week)			
	Never	0.84 (0.66–1.07)	0.81 (0.68–0.96)*
	1 to 3	1.01 (0.79–1.29)	1.01 (0.84–1.21)
	4 to 7	1.00	1.00
**Variables related to intrinsic capacity**			
Do you use glasses or other assistive devices to aid with vision problems?			
	Yes	1.00	1.00
	No	0.64 (0.51–0.79)*	0.77 (0.66–0.90)*
Do you use any assistive devices to aid mobility?			
	Yes	1.00	1.00
	No	2.30 (1.26–4.19)*	2.15 (1.39–3.33)*

AADL: Advanced Activities of Daily Living; OR odds ratio; 95%CI: 95% confidence interval; WHO: World Health Organization.

* CI values that do not reach 1.0.

Source: Prepared by the author.

Among demographic variables, aged men were 7.68 times more likely to exhibit a high level of participation in AADL. Individuals aged 60 to 69 were 4.46 times more likely, and white aged individuals were 64% more likely than black ones to participate at high levels. Divorced individuals had a 57% higher likelihood compared to widowed individuals, and those in the Southeast region were 57% more likely than those in the Northeast. In socioeconomic terms, aged individuals with higher education were 36 and 10.62 times more likely to have intermediate and high levels of participation in AADL.

Regarding clinical variables, aged individuals in "very good" health were nearly 7 times more likely to exhibit a high level of participation, while those who had not experienced a fall in the past 12 months were 49% more likely to participate at high levels. Additionally, aged individuals without chronic diseases were more likely to demonstrate high and intermediate levels of AADL participation.

Regarding lifestyle, not consuming alcohol reduces the chances of having a high level of participation by 79%, while engaging in physical activity increases the chances by 69%. Not smoking daily increases the chances of having an intermediate level of participation by 31%, and consuming vegetables or fruits 4 to 7 times a week increases the chances by 56% for the intermediate level. On the other hand, not consuming processed sugary foods decreases the chances by 19% for the intermediate level.

Finally, regarding intrinsic capacity variables, not wearing glasses decreased the likelihood of a high level of participation by 36%, while not using a mobility aid doubled the chances of having a high level of AADL participation.

## DISCUSSION

The results highlighted low participation of aged Brazilians in AADLs. The analysis revealed that aged males, between 60 and 69 years, self-identified as white, lived in the Southeast, South, and Central-West regions, had higher education levels, rated their health positively, had no recent history of falls, were free of circulatory or endocrine diseases, maintained healthy consumption habits, and had autonomy in transportation were the most likely to engage in AADLs.

The finding of low participation among older people in AADLs is consistent with previous studies conducted in Brazil, including one from 2020, which also identified low participation in various AADLs^10^. Additionally, research conducted in other settings, such as in China by Zhang et al.^15^, also observed low participation in AADLs.

One possible explanation for this finding is the influence of the family and social environment, where older individuals may choose not to engage in activities due to social or cultural pressures. The lack of engagement may stem from influences within the family environment, often driven by an attempt at preservation or protection.

In many cultures, chronological age is often used as an automatic criterion for vulnerability, dependence, and limited capacity to contribute. The way older individuals perceive aging is closely linked to their health and longevity prospects, with these views being strongly influenced by society and, particularly, by the family unit^16^.

To promote healthy and inclusive aging, it is essential to combat negative stereotypes, such as ageism, and foster a mindset that values the diversity and capabilities of older individuals. This involves ensuring that older people have access to meaningful opportunities for social participation and autonomy in decision-making.

When it comes to factors associated with the performance of AADLs, this study identified associations at different levels. Initially, at the distal level, in terms of education, older adults with higher educational levels were more likely to perform AADLs. This finding corroborates Usnayo et al.^13^, who highlighted the negative impact of low education on access to services and participation in social and leisure activities in Brazil.

Education plays a multifaceted role in the lives of older adults, affecting not only access to information and opportunities but also the ability to actively engage in society. The association between lower educational levels and lower participation in AADL is a relevant aspect to consider when seeking to promote healthy aging. These results reinforce the need for approaches that address educational disparities within the older adult population.

Regarding the different regions of the country, this research identified a lower probability of involvement in AADL in the Northeast region compared to the Southeast, South, and Central-West regions. According to research conducted by Oliveira et al.^17^, aged individuals in more precarious socioeconomic situations tend to rely more on public transportation and health services, in addition to residing in areas with greater material deprivation and limited social infrastructure. As a result, they face obstacles that hinder their participation in social activities, which are a key aspect of AADL.

The intermediate level of analysis in this study highlights the context of physical activity among older adults. Those who engage in this practice show a greater propensity to perform AADL. A meta-analysis conducted by Lin et al.^18^ highlighted the protective effect of physical activity for successful aging, and other studies suggest that social activities are linked to healthy lifestyles, with physical activity emerging as a crucial factor^19,20^.

These findings reinforce the importance of physical activity as a high-impact preventive strategy for the challenges faced by aging. It can support functional independence and the performance of AADLs among older adults, underscoring the need to promote and encourage this practice as an integral part of health care for this population.

Regarding tobacco consumption, data from this study indicate that non-smoking aged individuals tend to engage more in AADL, corroborating similar findings by Storeng et al.^21^, who associated daily smoking with low social participation, one of the AADLs.

It is important to highlight that tobacco consumption increases the risk of serious diseases, such as cancer, as emphasized by the WHO^22^. In addition to its health impacts, smoking affects respiratory capacity, limiting the daily activities of older adults. This underscores the need to identify contributing factors and develop strategies to reduce harm and improve their ability to participate in AADLs.

Regarding eating habits, it is worth noting that aged people who regularly consumed vegetables or legumes demonstrated greater engagement in AADL, while those who avoided sweet foods were less likely to participate in these activities.

Lifestyle, including dietary habits, plays a crucial role in the health of older adults, directly influencing the prevention of age-related diseases and the maintenance of functionality^23^. Strategies to increase the consumption of vegetables and whole grains are effective in promoting longevity and the ability to participate in AADL^24^.

In this context of aged people's diet, it is crucial to consider the environment in which they live and the opportunities available. Given that Brazil is marked by socioeconomic inequalities, these disparities influence this population's access to food and must always be taken into account.

Finally, in the multivariate analysis at the proximal level, a greater presence of men in the high and intermediate levels of AADL was identified compared to women. This finding is corroborated by a study that also indicated a greater probability of involvement in AADL among aged men, especially in activities related to work^25^.

The greater male representation in these activities may be related to the traditional division of roles, where aged women often assume household responsibilities, while men have more time to dedicate to AADLs. This dynamic may also be linked to the nature of the activities identified in the different levels of AADL in this study, where high and intermediate levels are more associated with work, while the low level is more related to religious activities.

Regarding race/color, it was observed that aged white individuals are more likely to engage in AADL than black individuals. Research conducted in Brazil has highlighted that the black population faces disproportionate exposure to factors that affect health and quality of life, such as unfavorable socioeconomic conditions, precarious housing, and limited access to education^26^.

These challenges hinder participation in AADL and reflect the racial inequities prevalent in Brazilian society. Therefore, it is essential to implement public policies and healthcare measures that address these disparities, with the goal of improving access to education, equitable healthcare, and adequate living conditions, thereby promoting equal opportunities for older individuals across diverse ethnic and socioeconomic backgrounds.

In the context of marital status, this research found that widowed individuals, and especially single aged people, are less likely to engage in AADL. This pattern aligns with findings from previous studies, such as one conducted in India, which linked marital status to participation in social activities^27^.

In addition to the emotional aspect, a marital partner fulfills multiple supportive roles, including providing daily assistance, financial support, emotional companionship, and health monitoring^28^. These findings underscore the importance of aging policies and programs that address specific needs through support networks, taking into account the complexities of marital status and its emotional and social consequences.

Regarding the influence of diseases on AADL participation, it is noteworthy that aged individuals without circulatory or endocrine system diseases tend to participate more actively. Among circulatory diseases, Hypertension is most prominent, while Diabetes Mellitus (DM) is the most prevalent endocrine condition.

It is important to note that approximately 92% of aged individuals have at least one age-related disease, primarily hypertension and DM, both of which are recognized as factors that can negatively affect functional capacity^29,30^.

In Brazil, programs such as Hypertension and Diabetes (HIPERDIA), established by the Ministry of Health in 2001, aim to address chronic diseases in the aged population, with a focus on the prevention, diagnosis, treatment, and control of hypertension and DM^31^. These initiatives reflect the need for integrated approaches to reduce the negative impacts of chronic diseases on the quality of life of aged individuals.

A limitation of this study is the potential for reverse causality in some variables, which is common in cross-sectional studies, where exposure and effect occur simultaneously. Additionally, a lack of articles specifically addressing AADL as conceptualized in this research was identified, as many studies treat these activities separately within categories such as social participation, leisure, and work.

The results of this study reinforce the significant influence of demographic, socioeconomic, clinical, lifestyle-related factors, and intrinsic capacity on the performance of AADLs. In a broader context, these findings have the potential to enhance and strengthen public policies and health programs that promote healthy aging.

Investing in infrastructure tailored to the needs of older adults, promoting inclusion across all aspects, and providing social support are effective strategies for encouraging engagement in AADL. Furthermore, ongoing research is essential to deepen the understanding of the participation of Brazilian older adults in AADL.

Investments in infrastructure tailored to the needs of older adults, promoting inclusion in all aspects, and providing social support emerge as effective strategies to encourage older adults’ engagement in AADL. Additionally, continued research is essential to enhance the understanding of Brazilian older adults’ participation in AADL.
