# Supplementary material for: Prevalence of participation of Brazilian aged people in Advanced Activities of Daily Living and associated factors
Source: Rev Bras Epidemiol. 2024 Dec 16;27:e240070. doi: 10.1590/1980-549720240070 (PMC11654639; doi:10.1590/1980-549720240070)
Supplement: Supplementary file 1 [file 1980-5497-rbepid-27-e240070-Suppl01.pdf]

MATERIAL SUPLEMENTAR **RBEPID-2024-0194**

Quadro Supl. 1 - Descrição das variáveis dependentes referentes às Atividades Avançadas de Vida Diária da Pesquisa Nacional de Saúde 2019.

**Variável**	**Pergunta da PNS**	**Categorização**	**Código PNS**
Participação em atividades coletivas de religião	Nos últimos doze meses, com que frequência o(a) Sr(a) compareceu a atividades coletivas da sua religião ou de outra religião? (Sem contar com situações como casamento, batizado ou enterro)	1.Uma ou mais vezes no ano<br>2.Nenhuma vez	M01901
Participação em reuniões sociais para atividades físicas, recreativas ou artísticas.	Nos últimos doze meses, com que frequência o(a) Sr(a) se reuniu com outras pessoas para prática de atividades esportivas, exercícios físicos, recreativos ou artísticos?	1.Uma ou mais vezes no ano<br>2.Nenhuma vez	M01601
Condução de automóvel	Atualmente, o(a) Sr(a) dirige automóvel (inclusive táxi, aplicativos de transporte e similares)?	1.Sim<br>2.Não	O00101
Trabalho voluntário	Nos últimos doze meses, com que frequência o(a) senhor(a) fez trabalho voluntário não remunerado?	1.Uma ou mais vezes no ano<br>2.Nenhuma vez	M01801
Trabalho remunerado	Na semana de 21 a 27 de julho de 2019 (semana de referência), ___ trabalhou ou estagiou, durante pelo menos uma hora, em alguma atividade remunerada em dinheiro?	1.Sim<br>2.Não	E001
Participação de associações ou movimentos sociais	Nos últimos doze meses, com que frequência o(a) Sr(a) participou de reuniões de grupos como associações de moradores ou funcionários, movimentos sociais/ comunitários, centros acadêmicos ou similares?	1.Uma ou mais vezes no ano<br>2.Nenhuma vez	M01701

Tabela Supl. 1- Resultados de adequação e ajuste dos modelos testados para as Classes Latentes das Atividades Avançadas de Vida Diária das pessoas idosas brasileiras, 2023.

	Número de Classes				
**Critérios Estatísticos**	**2**	**3**	**4**	**5**	**6**
AIC	139.420.882	138.443.956	138.071.430	137.961.970	137.910.031
BIC	139.525.290	138.604.583	138.288.277	138.235.036	138.239.317
BIC ajustado	139.483.976	138.541.024	138.202.472	138.126.985	138.109.020
Entropia	0.496	0.623	0.471	0.524	0.550
LRT Vuong-Lo-Mendell-Rubin	p=0.0000	p=0.0144	p=0.1962	p=0.1324	p=0.6941
LRT Lo-Mendell-Rubin	p=0.0000	p=0.0152	p=0.2007	p=0.1339	p=0.6958

Legenda: AIC= Critério de Informação de Akaike; BIC= Critério de Informação Bayseano; LRT= Teste de Verossimilhança

Fonte: Elaborado pela autora

Gráfico Supl. 1 – Discriminação entre as três classes a partir da frequência de participação das pessoas idosas nas Atividades Avançadas de Vida Diária, 2023.

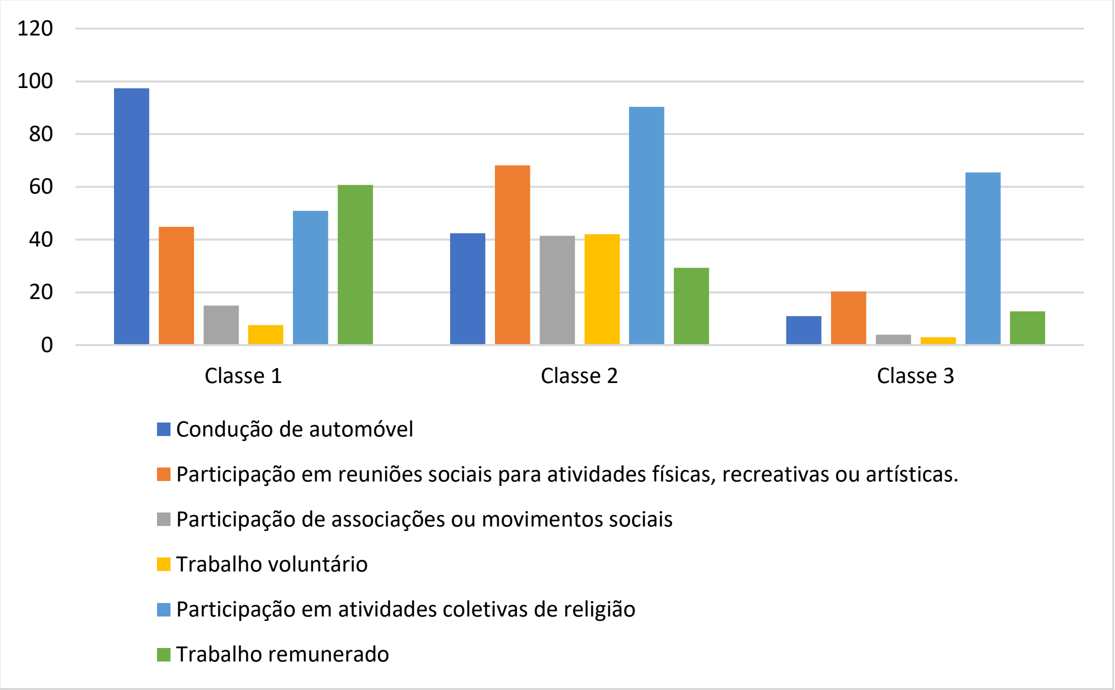

Fonte: elaborado pela autora
