# Supplementary material for: Prevalence of participation of Brazilian aged people in Advanced Activities of Daily Living and associated factors
Source: Rev Bras Epidemiol. 2024 Dec 16;27:e240070. doi: 10.1590/1980-549720240070 (PMC11654639; doi:10.1590/1980-549720240070)
Supplement: Translation

# Translation: Prevalência da participação de pessoas idosas brasileiras em Atividades Avançadas da Vida Diária e fatores associados

**DOI:** 10.1590/1980-549720240070.2

**Authors:** Vanessa de Barros e Silva Mazer, Rafael da Silveira Moreira, Kenio Costa de Lima, Maria das Graças Wanderley de Sales Coriolano, Vanessa de Lima Silva

**Affiliations:** I Universidade Federal de Pernambuco – Recife (PE), Brasil.; II Universidade Federal do Rio Grande do Norte – Natal (RN), Brasil.

**Keywords:** Atividades cotidianas, Idoso, Atividades de lazer, Participação social, Trabalho, Estado funcional

## Abstract

**Objetivo:**

Estimar a prevalência da participação de pessoas idosas brasileiras em Atividades Avançadas da Vida Diária e fatores associados.

**Métodos:**

Estudo transversal desenvolvido com dados secundários da Pesquisa Nacional de Saúde (PNS). Foram incluídas pessoas idosas (60 anos ou mais). A variável dependente foi composta pelas questões da PNS referentes à realização de Atividades Avançadas da Vida Diária relacionadas a trabalho, lazer e participação social. Foram realizados análise descritiva, teste de Rao-Scott e análise de resíduo padronizado. As medidas de efeito foram calculadas por modelos simples e múltiplos de regressão logística multinominal.

**Resultados:**

A maioria das pessoas idosas brasileiras apresenta baixa participação para as Atividades Avançadas da Vida Diária. As pessoas idosas do sexo masculino, entre 60 e 69 anos, brancas, das regiões Sudeste, Sul e Centro-Oeste, com níveis educacionais mais elevados, ausência de doenças crônicas, consumo mensal de álcool, prática de atividade física regular, ingestão frequente de verduras ou legumes, uso de óculos e independência no deslocamento estavam mais propensas a executar Atividades Avançadas da Vida Diária.

**Conclusão:**

Esses resultados reforçam a influência significativa dos fatores demográficos, socioeconômicos e clínicos ligados ao estilo de vida e à capacidade intrínseca na execução das Atividades Avançadas da Vida Diária. No âmbito mais amplo, tais descobertas podem fortalecer as políticas públicas de envelhecimento ativo e saudável.

## INTRODUÇÃO

A realização das Atividades da Vida Diária (AVD) é fundamental para conservação das capacidades físicas, mentais e sociais da pessoa idosa, funcionando como um poderoso parâmetro para avaliar a funcionalidade^1,2^. As AVD são, em Atividades Básicas da Vida Diária (ABVD), Atividades Instrumentais da Vida Diária (AIVD) e Atividades Avançadas da Vida Diária (AAVD)^3^.

As AAVD referem-se a funções físicas e sociais realizadas voluntariamente no cotidiano. Embora não sejam exclusivas a pessoas idosas, essa faixa etária tende a sofrer um maior declínio nessas atividades devido a alterações na saúde geral, bem como em aspectos cognitivos e sociais^4^.

Em 1989, Reuben e Solomon agruparam as AAVD em atividades relacionadas a integração social, atividades produtivas e atividades de lazer^5^. O comprometimento nessas atividades pode antecipar comprometimentos funcionais em AIVD e ABVD^6^.

A realização das AAVD está diretamente relacionada ao envelhecimento saudável, que envolve o desenvolvimento e a manutenção da capacidade funcional, contribuindo para o bem-estar na velhice^7,8^. Dessa forma, manter a independência funcional até o final da trajetória de vida é um dos principais objetivos, e o estado funcional surge como o novo paradigma para conhecer a saúde da pessoa idosa^9^.

Embora as AAVD pareçam familiares, na literatura são tratadas de diferentes maneiras. Muitos autores não diferenciam AAVD e AIVD, tratando todas como AVD. Já outros discutem as AAVD separadamente, dividindo-as em atividades de lazer, atividades de participação social e atividades relacionadas ao trabalho.

No contexto brasileiro, além dessas lacunas, observa-se a carência de análise dos fatores que influenciam a realização das AAVD. Um estudo realizado no Brasil em 2020 avaliou a quantidade de AAVD que as pessoas idosas realizavam em uma lista de 13 itens^10^. Já outra pesquisa, desenvolvida por Castro et al.^11^, avaliou o engajamento de pessoas idosas brasileiras em atividades laborais remuneradas.

Um estudo conduzido no Brasil em 2021 focou na participação de pessoas idosas em atividades sociais^12^. Usnayo et al.^13^, também no Brasil, identificaram fatores que interferem negativamente na participação das pessoas idosas em atividades sociais e de lazer.

O entendimento sobre os fatores que podem influenciar as AAVD em pessoas idosas brasileiras ainda é incipiente na literatura, visto que a maioria dos estudos foram realizados com amostras locais ou com as AAVD de forma isolada (lazer, trabalho ou participação social). Assim, é essencial investigar quais fatores impactam a realização dessas atividades.

Nesse cenário, esta pesquisa é relevante, pois ajuda a preencher a lacuna na literatura brasileira sobre AAVD. Além disso, tem um grande impacto científico, pois traz um panorama da capacidade funcional das pessoas idosas do Brasil antes da pandemia da COVID-19, servindo de base para comparação com pesquisas futuras.

Diante disso, o objetivo deste estudo consiste na identificação da prevalência da participação de pessoas idosas brasileiras em Atividades Avançadas da Vida Diária e fatores associados através de dados da Pesquisa Nacional de Saúde (PNS) de 2019.

## MÉTODOS

Foi realizado um estudo do tipo transversal de base populacional com abordagem analítica de dados secundários presentes no banco de dados da PNS de 2019. A população-alvo da PNS é constituída pelos moradores em domicílios particulares permanentes pertencentes a todo o território nacional. Para o presente estudo foram utilizados os dados da PNS referentes à população de pessoas idosas com 60 anos ou mais.

Em relação à amostragem, o plano amostral empregado pela PNS é o de amostragem conglomerada em três estágios, com estratificação das Unidades Primárias de Amostragem (UPAs). As primeiras UPAs são referentes aos setores censitários ou ao conjunto de setores, as segundas UPAs são os domicílios e as terceiras UPAs são os moradores de 15 anos ou mais de idade. A seleção de cada um dos três estágios foi feita por Amostragem Aleatória Simples (AAS).

O banco de dados da PNS-IBGE é público, está disponível no site do IBGE (http://www.ibge.gov.br) e foi salvo e organizado em um programa estatístico para análise dos dados. O projeto da PNS 2019 foi aprovado pela Comissão Nacional de Ética em Pesquisa (CONEP), do Conselho Nacional de Saúde (CNS), em agosto de 2019 sob o número 3.529.376 e obedeceu à Resolução 196/96 do Conselho Nacional de Saúde.

A variável dependente deste estudo corresponde às Atividades Avançadas de Vida Diária. Foi utilizada como base para a escolha das variáveis a lista de AAVD, constituída por atividades de âmbito social, produtivo e de lazer, aplicada pelo estudo Fragilidade em Idosos Brasileiros (FIBRA)^14^.

As variáveis relacionadas às AAVD foram selecionadas no banco de dados da PNS tendo como base os itens da lista de AAVD do protocolo FIBRA, sendo encontradas seis variáveis que se adequaram às AAVD: participação em atividades coletivas de religião; participação em reuniões sociais para atividades físicas, recreativas ou artísticas; condução de automóvel; trabalho voluntário; trabalho remunerado e participação de associações ou movimentos sociais (Quadro Suplementar 1).

Considerando que o fenômeno das AAVD não possui uma escala validada para mensurar seus resultados e que no questionário da PNS existem seis questões referentes às AAVD, a análise foi realizada por meio do método estatístico de Análise de Classes Latentes (ACL).

As variáveis independentes foram classificadas como variáveis demográficas (sexo, idade, raça/cor, estado civil e regiões); socioeconômicas (escolaridade e local de moradia); clínicas (autoavaliação da saúde segundo a Organização Mundial de Saúde – OMS, quedas, depressão, câncer, doença renal crônica, doenças do sistema circulatório, doenças do sistema endócrino, doenças do sistema respiratório e doenças do sistema osteomuscular); relacionadas ao estilo de vida (consumo de álcool, atividade física, uso de tabaco, consumo de verduras ou legumes, consumo de frutas, consumo de refrigerantes e consumo de alimentos doces industrializados); e relacionadas à capacidade intrínseca (visão, audição e locomoção).

Na ACL foi identificada a quantidade de classes que melhor definem o objeto de estudo, levando em consideração alguns critérios estatísticos: a entropia, o Critério de Informação Akaike (AIC), o Critério de Informação Bayesiano (BIC) e BIC ajustado, utilizados para avaliar os ajustes do modelo através do programa estatístico Mplus 8.

Para avaliar a evolução do modelo de testagem, os critérios Vuong, Lo, Mendell, Rubin — teste de verossimilhança (VLMR) e Lo, Mendell, and Rubin — teste de verossimilhança (LMR) foram utilizados considerando os valores de p<0,05 estatisticamente significantes. Para este estudo foram testados cinco modelos, com duas, três, quatro, cinco e seis classes latentes.

Os dados foram analisados com um programa estatístico que considerou os pesos e estratos amostrais da PNS. Variáveis com mais de cinco categorias foram reorganizadas. A análise descritiva incluiu frequência absoluta e relativa, sendo calculado o intervalo de confiança (IC) de 95%. Em seguida, foi analisada a presença de associação entre as variáveis independentes com a variável dependente por meio do teste de Rao-Scott utilizado em amostras complexas e análise de resíduo padronizado. O nível de significância adotado foi de 5%.

As medidas de efeito dos fatores sobre a variável dependente foram calculadas usando modelos simples e múltiplos de regressão logística multinominal, seguindo o princípio da abordagem hierarquizada de Victora. Para estratificar os níveis hierárquicos, utilizou-se o modelo de Determinantes Sociais da Saúde (DSS) de Dahlgren e Whitehead. Foram eleitas para análise múltipla as variáveis que obtiverem p<0,25 na análise simples.

## RESULTADOS

Dos 43.554 idosos do banco de dados da PNS, apenas 22.728 responderam ao questionário do morador e foram incluídos neste estudo. As frequências das variáveis independentes estão descritas na Tabela 1. A maioria das pessoas idosas eram do Nordeste (34%). A faixa etária predominante foi de 60 a 69 anos (55,2%), com 55,2% sendo do sexo feminino e 43,8% casados. A maioria residia em casa (83,3%), possuía ensino fundamental completo (48,9%) e se autodeclararam pardos (44%) ou brancos (43,6%).

**Tabela 1:** Análise descritiva e analítica das Atividades Avançadas de Vida Diária segundo blocos de variáveis demográficas, socioeconômicas, clínicas, relacionadas ao estilo de vida e à capacidade intrínseca. Pesquisa Nacional de Saúde, 2023.

Variável		Nível alto de participação em AAVD% (IC95%)	Nível intermediário de participação em AAVD% (IC95%)	Nível baixo de participação em AAVD% (IC95%)	Valor de p	Total %
**Variáveis demográficas**						
Sexo						
	Homem	82 (79–84,6)^a^	49,9 (47,6–52,1)^a^	37,6 (36,4–38,8)	<0,001*	44,8
	Mulher	18 (15,4–21)	50,1 (47,9–52,4)	62,4 (61,2–63,6)^a^		55,2
Idade (anos)						
	60 a 69	75 (72–77,8)^a^	66,7 (64,4–68,9)^a^	48,7 (87,5–49,9)	<0,001*	55,2
	70 a 79	21,4 (18,7–24,3)	26,9 (24,9–29,1)	33,6 (32,5–34,7)^a^		31,5
	80 ou +	3,6 (2,1–5,1)	6,4 (5,4–7,5)	17,7 (16,7–18,7)^a^		13,3
Raça/cor						
	Branco	68 (64,7–71,3)^a^	60,4 (58–62,6)^a^	47,5 (46,2–48,8)	<0,001*	43,6
	Negro	29,2 (26,3–32,4)	38 (35,8–40,3)	50,7 (49,4–52)^a^		54,8
	Amarelo/indígena	2,7 (1,7–4,1)	1,7 (1,1–2,5)	1,8 (1,5–2,2)		1,6
Estado civil						
	Casado(a)	59,7 (55,6–63,6)^a^	52,4 (50,1–54,7)^a^	38,6 (37,5–39,8)	<0,001*	43,8
	Divorciado(a)	17,8 (14,6–21,5)^a^	12,6 (11,2–14,2)	10,3 (9,6–11,1)		10,9
	Viúvo(a)	9,8 (8–12,1)	21,6 (19,7–23,6)	33 (31,8–34,1)^a^		26,8
	Solteiro(a)	12,7 (10,7–15)	13,4 (11,9–14,9)	18,1 (17,1–19)^a^		18,5
Regiões						
	Norte	3,1 (2,6–3,8)	4,1 (3,7–4,7)	5,7 (5,4–6)^a^	<0,001*	15,3
	Nordeste	13,6 (11,7–15,7)	16,6 (15,2–18,2)	27,6 (26,7–28,6)^a^		34
	Sudeste	56,6 (53,1–60,1)	47,2 (44,7–49,7)^a^	46,6 (45,4–47,9)		25,6
	Sul	19,7 (17,4–22,2)^a^	25,1 (23,2–27,1)^a^	14,4 (13,7–15,2)		14,6
	Centro-Oeste	7 (6–8,2)	7 (6,1–7,9)	5,6 (5,3–6)		10,4
**Variáveis socioeconômicas**						
Escolaridade						
	Sem instrução	0,2 (0,0–0,7)	0,1 (0,0–0,2)	0,6 (0,5–0,8)^a^	<0,001*	0,6
	Alfabetizado	0,9 (0,5–1,6)	2,8 (2,2–3,7)	6,6 (6–7,2)^a^		6,1
	Ensino fundamental	42,8 (39,4–46,2)	43,8 (41,1–46,4)	68,1 (66,8–69,4)^a^		48,9
	Ensino médio	24,7 (21,8–28)^a^	23,6 (21,5–25,7)^a^	16,5 (15,5–17,5)		15,7
	Ensino superior	31,4 (27,9–35,1)^a^	29,8 (27,3–32,3)^a^	8,2 (7,4–9)		11,8
Local de moradia						
	Casa	77,4 (73,2–81,1)	78,7 (76,3–80,9)	90,5 (89,6–91,4)^a^	<0,001*	88,3
	Apartamento	22,5 (18,8–26,7)^a^	22,2 (19–23,6)^a^	9,4 (8,5–10,3)		11,5
	Habitação em casa de cômodos, cortiço ou cabeça de porco	0,1 (0,0–0,2)	0,1 (0,0–0,3)	0,1 (0,0–0,1)		0,2
**Variáveis clínicas**						
Autoavaliação da saúde segundo a OMS						
	Muito boa	18,4 (15,8–21,2)^a^	19,6 (17,7–21,6)^a^	8,4 (7,7–9,2)	<0,001*	10,3
	Boa	60,3 (56,6–64)^a^	58 (55,7–60,2)^a^	48,4 (47,2–49,6)		51,4
	Regular	19,1 (16,6–21,9)	19,8 (18,1–21,7)	34,3 (33,1–35,6)^a^		30,9
	Ruim	2,1 (1,2–3,5)	2,4 (1,8–3,2)	6,9 (6,3–7,5)^a^		6,1
	Muito ruim	0,1 (0,0–0,7)	0,3 (0,1–0,6)	2 (1,7–2,4)^a^		1,3
Quedas						
	Sim	7,3 (5,8–9,1)	12,1 (10,8–13,6)	18,4 (17,5–19,4)^a^	<0,001*	16,4
	Não	92,7 (90,9–94,2)^a^	87,9 (86,4–89,2)^a^	81,6 (80,6–82,5)		83,6
Depressão						
	Sim	8,5 (6,4–11,1)	12,1 (10,7–13,6)	12,5 (11,7–13,3)^a^	0,011	10,4
	Não	91,5 (88,9–93,6)^a^	87,9 (86,4–89,3)	87,5 (86,7–88,3)		89,6
Câncer						
	Sim	6,6 (4,8–9,1)	8,8 (7,5–10,2)^a^	6,6 (6–7,2)	0,21	6,2
	Não	93,4 (90,9–95,2)	91,2 (89,8–92,5)	93,4 (92,8–94)^a^		93,8
Doença renal crônica						
	Sim	1,2 (0,8–2)	2,9 (2,2–3,9)^a^	2,4 (2,1–2,8)	0,23	2,3
	Não	98,8 (98–99,2)^a^	97,1 (96,1–97,8)	97,6 (97,2–97,9)		97,7
Doenças do sistema circulatório						
	Nenhuma	51,1 (47,9–54,3)^a^	45,7 (43,2–48,2)^a^	36,1 (35–37,3)	<0,001*	40,5
	1	40,9 (37,7–44,1)	44,6 (42,2–47)	49,3 (48,1–50,5)^a^		46,8
	2 ou 3	8,1 (6,3–10,2)	9,7 (8,4–11,2)	14,5 (13,7–15,4)^a^		12
Doenças do sistema endócrino						
	Nenhuma	67,5 (64,2–70,7)^a^	60,4 (57,9–62,9)	58,9 (57,7–60,2)	<0,001*	59,4
	1	26,7 (23,8–29,8)	33,7 (31,3–36,1)^a^	31,5 (30,4–32,7)		28,4
	2	5,8 (4,4–7,6)	5,9 (5–7,1)	9,5 (8,9–10,3)^a^		8,1
Doenças do sistema respiratório						
	Nenhuma	94,4 (92,7–95,8)	93,8 (92,7–94,8)	92,8 (92,1–93,5)	0,135	93,9
	1	5,1 (3,8–6,8)	5,3 (4,4–6,3)	6,1 (5,5–6,9)^a^		5,4
	2	0,4 (0,2–1)	0,9 (0,6–1,4)	1 (0,8–1,3)^a^		0,8
Doenças do sistema osteomuscular						
	Nenhuma	71,4 (67,6–74,9)^a^	61,2 (58,9–63,5)	59,5 (58,2–60,7)	<0,001*	62,3
	1	24,6 (21,5–28)	30,8 (28,7–33)	29,4 (28,2–30,5)		28,5
	2	4 (2,8–5,6)	8 (6,9–9,3)	11,2 (10,4–12)^a^		9,2
**Variáveis relacionadas ao estilo de vida**						
Consumo de álcool						
	Não bebo nunca	42,2 (38,5–46)	58,2 (55,9–60,6)	80,4 (79,3–81,4)^a^	<0,001*	75,2
	Menos de uma vez por mês	10,1 (8,4–12,2)^a^	11,2 (9,9–12,7)^a^	7 (6,4–7,7)		8,3
	Uma vez ou mais por mês	47,7 (43,7–51,7)^a^	30,6 (28,4–32,8)^a^	12,6 (11,8–13,5)		16,5
Atividade física						
	Sim	42,9 (39,5–46,4)^a^	59 (56,7–61,4)^a^	22,1 (21,2–23,1)	<0,001*	29,1
	Não	57,1 (53,6–60,5)	41 (38,6–43,3)	77,9 (76,9–78,8)^a^		70,9
Uso de tabaco						
	Sim, diariamente	14,6 (11,9–17,9)^a^	8,3 (7,1–9,6)	11,6 (10,9–12,4)	<0,001*	11,1
	Sim, menos que diariamente	0,3 (0,1–0,7)	0,4 (0,2–0,8)	0,7 (0,5–1)^a^		0,7
	Não fumo atualmente	85,1 (81,9–87,8)	91,3 (90–92,5)^a^	87,7 (86,8–88,4)		88,2
Consumo de verduras ou legumes (vezes por semana)						
	Nunca	3 (2,1–4,2)	2,4 (1,7–3,2)	6,7 (6,2–7,2)^a^	<0,001*	7,6
	1 a 3	20,2 (17,7–22,9)	19,2 (17,3–21,1)	26,7 (25,6–27,8)^a^		28,4
	4 a 7	76,8 (74–79,5)^a^	78,5 (76,4–80,4)^a^	66,6 (65,5–67,7)		64
Consumo de frutas (vezes por semana)						
	Nunca	7,8 (5,9–10,2)	4,7 (3,9–5,8)	7,9 (7,3–8,6)^a^	<0,001*	7,9
	1 a 3	23,6 (20,8–26,7)	20,2 (18,2–22,3)	28,7 (27,6–29,8)^a^		28,8
	4 a 7	68,6 (65,2–71,8)^a^	75,1 (72,8–77,2)	63,4 (62,2–64,5)		63,4
Consumo de refrigerantes (vezes por semana)						
	Nunca	62,1 (58,8–65,4)	69,5 (67,2–71,7)	70,3 (69,1–71,5)^a^	<0,001*	72,4
	1 a 3	28,5 (25,5–31,6)^a^	24,4 (22,4–26,5)	23,6 (22,5–24,7)		22,4
	4 a 7	9,4 (7,6–11,5)^a^	6,1 (5–7,3)	6,1 (5,5–6,9)		5,2
Consumo de alimentos doces industrializados (vezes por semana)						
	Nunca	47,6 (44,2–50,9)	48,4 (45,9–50,8)	56,5 (55,2–57,7)^a^	<0,001*	58,2
	1 a 3	34,5 (31,2–38)^a^	34,4 (32,1–36,8)^a^	28,7 (27,6–29,8)		28,1
	4 a 7	17,9 (15,2–21)^a^	17,3 (15,5–19,1)	14,9 (14–15,8)		13,6
**Variáveis relacionadas à capacidade intrínseca**						
Usa óculos ou outro aparelho de auxílio para lidar com problemas de visão?						
	Sim	82,2 (79,6–84,6)^a^	82,5 (80,7–84,2)^a^	70 (68,9–71,1)	<0,001*	69,9
	Não	17,8 (15,4–20,4)	17,5 (15,8–19,3)	30 (28,9–31,1)^a^		30,1
Usa aparelho auditivo ou outro aparelho de auxílio para ouvir melhor?						
	Sim	3,5 (2,4–5,1)	2,9 (2,2–3,8)	3,2 (2,8–3,7)	0,68	2,8
	Não	96,5 (94,9–97,6)	97,1 (96,2–97,8)	96,8 (96,3–97,2)		97,2
Usa algum aparelho de auxílio para se locomover?						
	Sim	1,6 (0,9–2,6)	1,9 (1,4–2,7)	8,6 (7,9–9,3)^a^	<0,001*	6,2
	Não	98,4 (97,4–99,1)^a^	98,1 (97,3–98,6)^a^	91,4 (90,7–92,1)		93,8

AAVD: Atividades Avançadas de Vida Diária; IC95%: intervalo de confiança de 95%; OMS: Organização Mundial de Saúde.

* p<0,05 (Teste de Rao e Scott);

a Resíduos padronizados >1,96.

Fonte: Elaborado pela autora.

Quanto à saúde, 46,8% tinham doenças circulatórias e 37,7% osteomusculares, enquanto 83,6% não tinham caído nos últimos 12 meses. Na questão de estilo de vida, a maioria não consumia álcool (75,2%), não praticava atividade física (70,9%), não fumava (88,2%), consumia verduras ou legumes diariamente (64%) e frutas com a mesma frequência (63,4%). A maior parte não consumia refrigerantes (72,4%) ou doces industrializados (58,2%).

O modelo de três classes latentes foi escolhido pois apresentou melhor desempenho (Tabela Suplementar 1), revelando padrões distintos de engajamento em AAVD entre as classes identificadas.

Considerando a definição adotada para as AAVD nesta pesquisa, juntamente com a frequência de participação nas atividades e sua complexidade (Gráfico Suplementar 1), as seguintes categorias foram atribuídas: nível alto de participação para classe 1, nível intermediário de participação para classe 2 e nível baixo de participação para classe 3.

A maioria teve uma participação considerada baixa nas AAVD, com uma proporção significativa de 73,1%, seguido por uma participação intermediária (18,1%) e uma alta participação (8,9%).

A análise bivariada (Tabela 1) mostrou associações significativas entre a realização de AAVD e várias variáveis, como sexo, idade, raça/cor, estado civil, região, escolaridade, saúde autoavaliada, histórico de quedas, depressão e várias doenças crônicas. Outros fatores associados incluíram consumo de álcool, prática de atividade física, tabagismo, hábitos alimentares, uso de dispositivos de correção visual e auxílios para locomoção.

A análise de regressão logística multinominal (Tabela 2) foi conduzida considerando as variáveis que obtiveram valores de p<0,25 no teste de Rao Scott. Os fatores associados que permaneceram no modelo final, baseado nos níveis hierárquicos dos DSS, estão demonstrados na Figura 1.

**Tabela 2:** Valores de *odds ratio* ajustada e intervalos de confiança obtidos por análise de regressão logística multinominal para associação entre as variáveis demográficas, socioeconômicas, clínicas, relacionadas ao estilo de vida e à capacidade intrínseca e as Atividades Avançadas de Vida Diária, 2023.

Variável		Nível alto de participação em AAVD	Nível intermediário de participação em AAVD % (IC95%)
		OR (IC95%)	OR (IC95%)
**Variáveis demográficas**			
Sexo			
	Homem	7,68 (6,12–9,64)*	1,84 (1,59–2,12)*
	Mulher	1,00	1,00
Faixa etária (anos)			
	60 a 69	4,46 (2,89–6,88)*	2,25 (1,77–2,84)*
	70 a 79	2,14 (1,37–3,35)*	1,54 (1,21–1,96)*
	80 ou +	1,00	1,00
Raça/cor			
	Branco	1,64 (1,34–2,01)*	1,07 (0,93–1,23)
	Amarelo/indígena	1,77 (0,97–3,22)	0,78 (0,36–1,42)
	Negro	1,00	1,00
Estado civil			
	Casado(a)	1,25 (0,94–1,68)	1,14 (0,96–1,35)
	Divorciado(a)	1,57 (1,06–2,31)*	0,92 (0,73–1,16)
	Solteiro(a)	0,95 (0,66–1,36)	0,74 (0,59–0,92)*
	Viúvo(a)	1,00	1,00
Regiões			
	Norte	1,04 (0,81–1,33)	1,20 (1,00–1,45)
	Sudeste	1,57 (1,28–1,94)*	1,18 (1,01–1,37)*
	Sul	2,15 (1,74–2,64)*	2,55 (2,18–2,99)*
	Centro-oeste	1,96 (1,53–2,50)*	1,13 (1,44–2,08)*
	Nordeste	1,00	1,00
**Variáveis socioeconômicas**			
Escolaridade			
	Alfabetizado	0,53 (0,12–2,26)*	5,07 (1,48–17,31)*
	Ensino fundamental	1,71 (0,44–6,59)*	5,84 (1,76–19,34)*
	Ensino médio	4,1 (1,07–16,09)*	13,63 (4,1–45,27)*
	Ensino superior	10,62 (2,74–41,17)*	36,02 (10,82–119,82)*
	Sem instrução	1,00	1,00
**Variáveis clínicas**			
Autoavaliação da saúde segundo a OMS			
	Muito boa	6,37 (1,23–32,89)*	7,99 (3,02–21,24)*
	Boa	5,03 (0,99–25,61)	5,82 (2,20–15,40)*
	Regular	3,53 (0,69–18,07)	4,11 (1,55–10,86)*
	Ruim	2,96 (0,52–16,74)	4,08 (1,45–11,44)*
	Muito ruim	1,00	1,00
Quedas			
	Sim	1,00	1,00
	Não	1,49 (1,09–2,03)*	1,05 (0,88–1,25)
Doenças do sistema circulatório			
	Nenhuma	1,53 (1,12–2,11)*	1,21 (0,96–1,52)
	1	1,29 (0,94–1,76)	1,05 (0,85–1,31)
	2 ou 3	1,00	1,00
Doenças do sistema endócrino			
	Nenhuma	1,17 (0,80–1,71)	1,38 (1,07–1,78)*
	1	1,25 (0,86–1,83)	1,18 (0,93–1,50)
	2	1,00	1,00
**Variáveis relacionadas ao estilo de vida**			
Consumo de álcool			
	Não bebo nunca	0,21 (0,17–0,25)*	0,45 (0,39–0,53)*
	Menos de uma vez por mês	0,43 (0,33–0,57)*	0,75 (0,60–0,94)*
	Uma vez ou mais por mês	1,00	1,00
Atividade física			
	Sim	1,69 (1,43–2,00)*	3,65 (3,24–4,11)*
	Não	1,00	1,00
Uso de tabaco			
	Não fumo atualmente	0,87 (0,67–1,13)	1,31 (1,05–1,64)*
	Sim, menos que diariamente	0,38 (0,14–0,99)*	0,82 (0,30–2,22)
	Sim, diariamente	1,00	1,00
Consumo de verduras ou legumes (vezes por semana)			
	4 a 7	1,12 (0,76–1,67)	1,56 (1,07–2,27)*
	1 a 3	1,11 (0,73–1,68)	1,39 (0,93–2,08)
	Nunca	1,00	1,00
Consumo de alimentos doces industrializados (vezes por semana)			
	Nunca	0,84 (0,66–1,07)	0,81 (0,68–0,96)*
	1 a 3	1,01 (0,79–1,29)	1,01 (0,84–1,21)
	4 a 7	1,00	1,00
**Variáveis relacionadas à capacidade intrínseca**			
Usa óculos ou outro aparelho de auxílio para lidar com problemas de visão?			
	Sim	1,00	1,00
	Não	0,64 (0,51–0,79)*	0,77 (0,66–0,90)*
Usa algum aparelho de auxílio para se locomover?			
	Sim	1,00	1,00
	Não	2,30 (1,26–4,19)*	2,15 (1,39–3,33)*

AAVD: Atividades Avançadas de Vida Diária; OR *odds ratio*; IC95%: intervalo de confiança de 95%; OMS: Organização Mundial de Saúde.

* valores em que o IC não toca 1,0.

Fonte: Elaborado pela autora.

**Figura 1:** Modelo final das variáveis da regressão logística multinominal dos fatores associados à realização das Atividades Avançadas de Vida Diária em pessoas idosas brasileiras, 2023.

Fonte: Elaborado pela autora.

Nas variáveis demográficas, homens idosos têm 7,68 vezes mais chances de ter um alto nível de participação em AAVD. Pessoas de 60 a 69 anos têm 4,46 vezes mais chances; pessoas idosas brancas têm 64% mais chances do que as negras; divorciados têm 57% mais chances do que viúvos. Na região Sudeste, as chances são 57% maiores do que no Nordeste. Em termos socioeconômicos, pessoas idosas com ensino superior têm 36 e 10,62 vezes mais chances de terem níveis intermediário e alto de participação em AAVD.

Nas variáveis clínicas, ter saúde "muito boa" apresentou quase 7 vezes mais chances de ter um alto nível de participação e aquelas que não caíram nos últimos 12 meses têm 49% mais chances. Além disso, as pessoas idosas sem doenças têm mais chances de terem níveis alto e intermediário de participação em AAVD.

Quanto ao estilo de vida, não consumir álcool diminui as chances de ter um alto nível de participação em 79%, enquanto praticar atividade física aumenta em 69%. Não fumar diariamente aumenta em 31% as chances de ter um nível intermediário de participação e consumir verduras ou legumes de 4 a 7 vezes por semana aumenta as chances em 56% para o nível intermediário. Por outro lado, não consumir alimentos doces industrializados diminui as chances em 19% para o nível intermediário.

Finalmente, no âmbito das variáveis relacionadas à capacidade intrínseca, não usar óculos diminui as chances em 36% para o alto nível de participação, enquanto não usar aparelho de auxílio para locomoção aumenta as chances em 2 vezes para o alto nível de participação em AAVD.

## DISCUSSÃO

Os resultados destacaram uma baixa participação das pessoas idosas brasileiras em AAVD. A análise revelou que pessoas idosas do sexo masculino, com idade entre 60 e 69 anos, de autodeclaração branca, residentes nas regiões Sudeste, Sul e Centro-Oeste, com maior escolaridade, autoavaliação de saúde positiva, sem histórico recente de quedas, sem doenças circulatórias ou endócrinas, com hábitos de consumo saudáveis e autonomia no deslocamento foram as mais propensas a participar das AAVD.

A constatação da baixa participação das pessoas idosas em AAVD é consistente com estudos anteriores realizados no Brasil, incluindo um, conduzido em 2020, que também identificou a baixa participação de pessoas idosas em diferentes AAVD^10^. Além disso, pesquisas conduzidas em outros cenários, como na China, por Zhang et al.^15^, também identificaram baixa participação em AAVD.

Uma possível explicação para esse achado é a influência do ambiente familiar e social, em que as pessoas idosas podem optar por não se engajar em atividades devido a pressões sociais ou culturais. Existe a possibilidade de que a falta de engajamento ocorra devido a influências provenientes do ambiente familiar, muitas vezes relacionadas a uma tentativa de preservação.

Em diversas culturas, a idade cronológica frequentemente é utilizada como um critério automático de vulnerabilidade, dependência e capacidade de contribuição limitada. A percepção que as pessoas idosas têm em relação ao envelhecimento está diretamente relacionada às suas perspectivas de saúde e longevidade, e essas visões são fortemente influenciadas pela sociedade e, especialmente, pelo núcleo familiar^16^.

Para promover um envelhecimento saudável e inclusivo, é fundamental combater estereótipos negativos, como o etarismo, e criar uma mentalidade que valorize a diversidade e a capacidade das pessoas idosas. Isso implica garantir que elas tenham acesso a oportunidades significativas de participação social e autonomia na tomada de decisões.

Quando se trata dos fatores associados à realização das AAVD, este estudo identificou associações em diferentes níveis. Inicialmente, no nível distal, no âmbito da escolaridade, pessoas idosas com nível educacional mais elevado apresentaram maior probabilidade de desenvolver as AAVD. Esse achado corrobora com Usnayo et al.^13^, que destacaram o impacto negativo da baixa escolaridade no acesso a serviços e na participação em atividades sociais e de lazer no Brasil.

A educação desempenha um papel multifacetado na vida das pessoas idosas, afetando não apenas o acesso a informações e oportunidades, mas também a capacidade de se envolver ativamente na sociedade. A associação entre menor nível educacional e menor participação em AAVD é um aspecto relevante a ser considerado quando se busca promover o envelhecimento saudável. Esses resultados reforçam a necessidade de abordagens que levem em consideração as disparidades educacionais na população de pessoas idosas.

No que concerne às diferentes regiões do país, esta pesquisa identificou uma menor probabilidade de envolvimento em AAVD na região Nordeste em comparação com as regiões Sudeste, Sul e Centro-Oeste. De acordo com a pesquisa realizada por Oliveira et al.^17^, pessoas idosas em situações socioeconômicas mais precárias tendem a depender mais de serviços de transporte público e de saúde, além de viverem em áreas associadas a maiores privações materiais e de infraestrutura social. Dessa maneira, enfrentam obstáculos que limitam sua participação em atividades sociais, uma das AAVD.

O nível intermediário da análise deste estudo destaca o contexto da atividade física entre as pessoas idosas. Aquelas que se engajam nessa prática demonstraram maior propensão para realizar AAVD. Uma meta-análise realizada por Lin et al.^18^ destacou o efeito protetor da atividade física para um envelhecimento bem-sucedido e outros estudos sugerem que atividades sociais estão ligadas a estilos de vida saudáveis, com a atividade física emergindo como um fator crucial^19,20^.

Esses achados reforçam a vitalidade da atividade física como uma estratégia preventiva de grande impacto para os desafios enfrentados pelo envelhecimento. Ela pode sustentar a independência funcional e a realização de AAVD entre as pessoas idosas, reforçando a necessidade de promover e incentivar essa prática como parte integral do cuidado à saúde dessa população.

No tocante ao consumo de tabaco, os dados deste estudo indicam que as pessoas idosas não fumantes tendem a se envolver mais em AAVD, corroborando achados similares de Storeng et al.^21^, que associaram o tabagismo diário à baixa participação social, uma das AAVD.

É importante destacar que o consumo de tabaco aumenta o risco de doenças graves, como câncer, conforme ressaltado pela OMS^22^. Além dos impactos na saúde, o tabagismo afeta a capacidade respiratória, limitando as atividades diárias das pessoas idosas, destacando a necessidade de identificar fatores contribuintes e desenvolver estratégias para reduzir danos e melhorar sua capacidade de participação.

No que diz respeito aos hábitos alimentares, destaca-se que as pessoas idosas que consumiam verduras ou legumes regularmente demonstraram maior engajamento em AAVD, enquanto aquelas que evitavam alimentos doces tinham menor probabilidade de desenvolvê-las.

O estilo de vida, incluindo os hábitos alimentares, desempenha um papel crucial na saúde das pessoas idosas, influenciando diretamente a prevenção de doenças relacionadas ao envelhecimento e à manutenção da funcionalidade^23^. Estratégias para aumentar o consumo de legumes e grãos integrais são eficazes para promover a longevidade e a capacidade de participar das AAVD^24^.

Nesse contexto alimentar das pessoas idosas, é crucial considerar o ambiente em que estão inseridas e as oportunidades disponíveis. Dado que o Brasil é marcado por desigualdades socioeconômicas, essas disparidades influenciam o acesso dessa população à alimentação e devem ser sempre ponderadas.

Por último, na análise multivariada do nível proximal, inicialmente, em relação ao sexo, identificou-se maior presença de homens nos níveis alto e intermediário de AAVD em comparação com as mulheres. Essa constatação é corroborada por uma pesquisa que também apontou uma maior probabilidade de envolvimento em AAVD entre homens idosos, especialmente aquelas relacionadas ao trabalho^25^.

A maior representatividade masculina nessas atividades pode estar relacionada à divisão tradicional de papéis, onde as mulheres idosas frequentemente assumem responsabilidades domésticas, enquanto os homens têm mais tempo para dedicar-se às AAVD. Essa dinâmica também pode estar relacionada à natureza das atividades encontradas nos diferentes níveis de AAVD identificados neste estudo, em que os níveis alto e intermediário estão mais ligados ao trabalho, enquanto o nível baixo está mais relacionado a atividades religiosas.

Em relação à raça/cor foi possível observar que as pessoas idosas de cor branca têm uma maior propensão a se envolverem em AAVD do que as de raça negra. Pesquisas realizadas no Brasil destacaram que a população negra enfrenta exposição desproporcional a fatores que afetam a saúde e a qualidade de vida, como condições socioeconômicas desfavoráveis, habitação precária e menor acesso à educação^26^.

Esses desafios influenciam negativamente a capacidade de participar das AAVD, refletindo as iniquidades raciais enfrentadas pela sociedade brasileira. Diante disso, é crucial adotar políticas públicas e medidas de saúde que abordem essas disparidades, visando melhorar o acesso a educação, cuidados de saúde equitativos e condições de vida adequadas para promover a igualdade de oportunidades entre pessoas idosas de diferentes origens étnicas e socioeconômicas.

No contexto do estado civil, a presente pesquisa identificou que pessoas idosas viúvas e, principalmente, solteiras têm menor probabilidade de se envolverem em AAVD. Esse padrão é consistente com estudos anteriores, como o realizado na Índia, que associou o estado civil à participação em atividades sociais^27^.

Além do aspecto emocional, o parceiro conjugal desempenha múltiplos papéis de suporte, incluindo assistência diária, apoio financeiro, companheirismo emocional e monitoramento da saúde^28^. Esses achados destacam a importância de políticas e programas de envelhecimento que atendam às necessidades específicas por meio de redes de apoio sensíveis às complexidades do estado civil e suas consequências emocionais e sociais.

No que tange à influência das doenças na realização das AAVD, é notável que as pessoas idosas desprovidas de doenças do sistema circulatório e endócrino tendem a apresentar uma maior participação. Entre as doenças circulatórias, a Hipertensão Arterial Sistêmica (HAS) ganha destaque, enquanto, no âmbito das doenças endócrinas, a Diabetes Mellitus (DM) prevalece.

É importante ressaltar que cerca de 92% das pessoas idosas possuem pelo menos uma doença relacionada à idade, principalmente HAS e DM, e que elas são reconhecidas como fatores que podem impactar negativamente a capacidade funcional^29,30^.

No Brasil, programas como o Hipertensão e Diabetes (HIPERDIA), estabelecido pelo Ministério da Saúde em 2001, visam enfrentar as doenças crônicas na população idosa, com foco na prevenção, no diagnóstico, tratamento e controle da HAS e DM^31^. Essas iniciativas refletem a necessidade de abordagens integradas para mitigar os impactos negativos das doenças crônicas na qualidade de vida das pessoas idosas.

Uma limitação deste estudo foi a possibilidade de causalidade reversa em algumas variáveis, comum em estudos transversais, em que exposição e efeito ocorrem simultaneamente. Além disso, foi identificada a escassez de artigos que abordassem especificamente as AAVD conforme conceituadas nesta pesquisa, já que muitos estudos as tratam separadamente em categorias como participação social, lazer e trabalho.

Os resultados do presente estudo reforçam a influência significativa dos fatores demográficos, socioeconômicos, clínicos, ligados ao estilo de vida e à capacidade intrínseca na execução das AAVD. No âmbito mais amplo, tais descobertas têm potencial para a otimização e o fortalecimento das políticas públicas e programas de saúde que promovem o envelhecimento saudável.

Investir em infraestrutura adaptada às necessidades das pessoas idosas, promover a inclusão em todos os aspectos e oferecer suporte social são estratégias eficazes para fomentar o engajamento em AAVD. Além disso, a continuidade das pesquisas é crucial para aprofundar o conhecimento sobre a participação das pessoas idosas brasileiras em AAVD.

Investimentos em infraestrutura adaptada às necessidades das pessoas idosas, incentivo à inclusão em todos os aspectos e fornecimento de apoio social emergem como estratégias eficazes para estimular o engajamento das pessoas idosas nas AAVD. Além disso, a continuidade de pesquisas é fundamental para enriquecer as informações referentes à participação de pessoas idosas brasileiras em AAVD.
